# Whole-Genome Sequencing of Suppressor DNA Mixtures Identifies Pathways That Compensate for Chromosome Segregation Defects in *Schizosaccharomyces pombe*

**DOI:** 10.1534/g3.118.200048

**Published:** 2018-01-19

**Authors:** Xingya Xu, Li Wang, Mitsuhiro Yanagida

**Affiliations:** G0 Cell Unit, Okinawa Institute of Science and Technology Graduate University, Onna-son, Okinawa 904-0495, Japan

**Keywords:** *Schizosaccharomyces pombe*, temperature sensitive mutant, Suppressor screen, next-generation sequencing, chromosome segregation

## Abstract

Suppressor screening is a powerful method to identify genes that, when mutated, rescue the temperature sensitivity of the original mutation. Previously, however, identification of suppressor mutations has been technically difficult. Due to the small genome size of *Schizosaccharomyces pombe*, we developed a spontaneous suppressor screening technique, followed by a cost-effective sequencing method. Genomic DNAs of 10 revertants that survived at the restrictive temperature of the original temperature sensitive (ts) mutant were mixed together as one sample before constructing a library for sequencing. Responsible suppressor mutations were identified bioinformatically based on allele frequency. Then, we isolated a large number of spontaneous extragenic suppressors for three ts mutants that exhibited defects in chromosome segregation at their restrictive temperature. Screening provided new insight into mechanisms of chromosome segregation: loss of Ufd2 E4 multi-ubiquitination activity suppresses defects of an AAA ATPase, Cdc48. Loss of Wpl1, a releaser of cohesin, compensates for the Eso1 mutation, which may destabilize sister chromatid cohesion. The segregation defect of a ts histone H2B mutant is rescued if it fails to be deubiquitinated by the SAGA complex, because H2B is stabilized by monoubiquitination.

Temperature-sensitive (ts) mutants are very useful to identify genes involved in specific biological processes, and to discover the functions of essential genes in model organisms such as *Schizosaccharomyces pombe* and *Saccharomyces cerevisiae*. Much of our understanding of cell division and chromosome segregation is derived from analysis of ts mutants in yeasts. In *S. pombe*, genes involved in chromosome segregation were isolated by screening for ts mutants that exhibited chromosome segregation defects or high rates of minichromosome loss at the restrictive temperature (Hirano *et al.* 1986; Samejima *et al.* 1993; Takahashi *et al.* 1994; Hayashi *et al.* 2004; Xu *et al.* 2015). Genes that are required for entry into and/or maintenance of quiescence were identified from a ts mutant collection after nitrogen starvation (Sajiki *et al.* 2009).

Isolation of extragenic suppressors is a powerful tool for identifying genes with protein products that function in the same process as a gene of interest, or that physically interact with the protein product of that gene (van Leeuwen *et al.* 2017). For example, a phosphatase deletion mutant, *Δppe1*, is cold-sensitive (cs) and staurosporine-hypersensitive (Shimanuki *et al.* 1993; Matsusaka *et al.* 1995). A protein kinase, Ssp1, that closely interacts with Ppe1 was isolated as a suppressor of cs or staurosporine sensitivity of *Δppe1* (Matsusaka *et al.* 1995). Cold-sensitive dominant mutants, *scn1* and *scn2*, of *S. pombe*, which have the same substitution in the anticodons of two tRNA-Ala (UGC) genes, were isolated by their ability to suppress the temperature-sensitivity of an anaphase-promoting complex/cyclosome (APC/C) mutant, *cut9-665* (Samejima and Yanagida 1994). Mutations in Ppe1 phosphatase and its bound partner, Ekc1, were isolated as a result of their ability to suppress the temperature-sensitivity of a kinetochore mutant, *mis12-537* (Goshima *et al.* 2003).

Since the *S. pombe* genome is very small (∼12.5 Mb), and next-generation sequencing is a high-throughput technique, we established a spontaneous suppressor screening method followed by sequencing of a mixture of revertants from the same *ts* mutant. By comparing with the classical genetic methods used to identify suppressor mutations in above studies, this approach does not need the revertants to be cold sensitive (cs) and does not need cloning the responsible suppressors by genetic complementation; therefore, it saves a lot of time and labor, at the same time it enables identification of multiple suppressor mutations simultaneously and quickly (2–3 months including genomic DNA preparation, library construction, next-generation sequencing, and suppressor mutation identification). Our approach allowed us to isolate and analyze extragenic suppressors for three *S. pombe* ts mutants defective in chromosome segregation at the restrictive temperature easily.

## Materials and Methods

### Strains, plasmids, and media

Fission yeast strains used in this study (Table 1) are derivatives of the wild-type heterothallic strains 972*h^−^* and 975*h^+^*. Temperature-sensitive mutants, *cdc48-353* (Ikai and Yanagida 2006), *eso1-H17* (Tanaka *et al.* 2000), and *htb1-72* (Maruyama *et al.* 2006) were selected for suppressor screening. Their ts mutations were reintegrated into the *S. pombe* haploid wild-type strain, 972 *h^−^* using site-directed, PCR-based mutagenesis. Briefly, complementary pairs of synthesized DNA oligos with ts mutations were used as PCR primers, followed by two rounds of PCR. Mutated genes (ORFs) were cloned and were ligated into pBluescript in upstream of a hygromycin-resistant antibiotic marker (hphMX4). Then, ∼500 bp long sequences after the corresponding ORFs were cloned and were ligated into the above plasmid downstream of the antibiotic marker. The plasmids were linearized and were chromosomally integrated into corresponding endogenous loci of the aforementioned 972 *h^−^* wild-type strain. Hygromycin-resistant colonies were picked up and then ts candidates, which could grow at 26° but could not grow at 36°, were selected. The ts mutations were confirmed by Sanger sequencing of the mutated genes. *Δufd2*, *Δubp8*, and *Δgcn5* strains were obtained from a purchased *S. pombe* haploid deletion mutant library (Bioneer Corporation). Rich complete plates or medium (YES plates or YES medium) were used for culturing these auxotrophic mutant strains. Other strains (972 *h^−^* wild-type strain, *cdc48-G338D*, *eso1-G799D*, and *htb1-G52D*) are prototrophic and were cultured on YPD plates or in YPD media. YES media and YES plates were used for spot test experiments.

**Table 1:** Fission yeast strains used in this study

Strain	Genotype	Reference
Wild type	972 *h^−^*	Laboratory stock
*cdc48-G338D*	*h^−^ cdc48-G338D*::*hphMX4*	This study
*eso1-G799D*	*h^−^ eso1-G799D*::*hphMX4*	This study
*htb1-G52D*	*h^−^ htb1-G52D*::*hphMX4*	This study
*Δufd2*	*h^+^ ura4-D18 leu1-32 Δufd2*::*KanMX4*	Bioneer
*Δubp8*	*h^+^ ura4-D18 leu1-32 Δubp8*::*KanMX4*	Bioneer
*Δgcn5*	*h^+^ ura4-D18 leu1-32 Δgcn5*::*KanMX4*	Bioneer
*cdc48-G338D Δufd2*	*h^−^ leu1-32 cdc48-G338D*::*hphMX4 Δufd2*::*KanMX4*	This study
*htb1-G52D Δubp8*	*h^−^ leu1-32 htb1-G52D*::*hphMX4 Δubp8*::*KanMX4*	This study
*htb1-G52D Δgcn5*	*h^−^ leu1-32 htb1-G52D*::*hphMX4 Δgcn5*::*KanMX4*	This study

### Suppressor screening strategy

Several different restrictive temperatures were tested for each ts mutant, according to the intensity of temperature-sensitivity (spot test results). Appropriate restrictive temperatures were then selected for each ts mutant to ensure revertant frequencies between 1 × 10^−8^ and 1 × 10^−6^. Ts mutants were cultured in YPD liquid medium at the permissive temperature (26°) overnight. To increase suppressor mutation complexity, multiple (eight for *cdc48-G338D*, twelve for *eso1-G799D* and nine for *htb1-G52D*) independent 50-ml conical tubes with 10 ml medium in each were used, and only ∼10% (or less) cells in each tube were plated onto YPD plates at a concentration of 1 × 10^6^–1 × 10^7^ cells per plate, followed by culturing at the selected restrictive temperature for 4 d. All colonies with reasonable size (revertants that contained the original ts mutations and suppressor mutations) were picked up for further analysis.

### DNA sequencing sample preparation, library preparation, and Illumina paired-end sequencing

Revertant cells from 20-ml YPD liquid cultures (30°; overnight) were collected and their genomic DNA was extracted using Maxwell 16 System DNA Purification Kits. Then, DNA concentration was measured using an Ultrospec 3100 *pro*. Since all revertants of the same ts mutant contain the same original ts mutation, and differ by only one suppressor mutation, we mixed equal amounts of genomic DNA of 10 revertants from the same ts mutant so as to create one genomic DNA mixture. This genomic DNA mixture was used for library construction. Each 10 DNA mixtures were barcoded for one-lane pooled sequencing. DNA libraries for Illumina sequencing were generated using standard protocols (Illumina) and were sequenced with paired-end (2 × 150 bp) runs using Illumina HiSequation 2000 sequencers.

### Alignment, mutation calling, and mutation filtering

Sequence reads were mapped against the *S. pombe* reference genome (downloaded from Pombase: https://www.pombase.org) using the Novoalign mapping tool (V3.00.02; http://www.novocraft.com) with default settings. Resulting SAM files were converted to BAM, and were then sorted and indexed with Samtools (V0.1.18; Li *et al.* 2009). Mutations were called by SNVer (V0.5.3; Wei *et al.* 2011). Since each mixture contains genomic DNA of 10 revertants, 10 (or fewer) suppressor mutations, in addition to the original ts mutation, should be identified, with suppressor allele frequencies at ∼10% and original ts mutation at 100%. Mutations identified by SNVer with allele frequency ≥2% were selected, and then genes with more than two independent mutations in them or more than two independent mutations in genes involved in same complex were manually selected as suppressor candidates for further analysis. Suppressor mutations were confirmed independently by Sanger sequencing.

### Immunoblotting

Ten milliliters of *S. pombe* cell culture (containing 1 × 10^8^ cells) was mixed with 1/4 vol (2.5 ml) of ice-cold 100% trichloroacetic acid (TCA). The resulting mixture was centrifuged, and pellets were washed with 10% TCA, followed by cell disruption with glass beads in 10% TCA. After centrifugation at 8000 rpm for 10 min at 4°, washed precipitates were resuspended in SDS sample buffer containing 1 mM phenylmethylsulfonyl fluoride (PMSF) and boiled at 70° for 10 min. After centrifugation at 14,000 rpm for 10 min, the supernatant was used for 12% Bis-Tris gels (MES buffer) and immunoblotted.

### Data availability

Illumina sequence reads have been deposited in the NCBI Sequence Read Archive under BioProject ID PRJNA415340. Strains are available upon request.

## Results

### Spontaneous suppressor identification strategy

**Figure 1:**
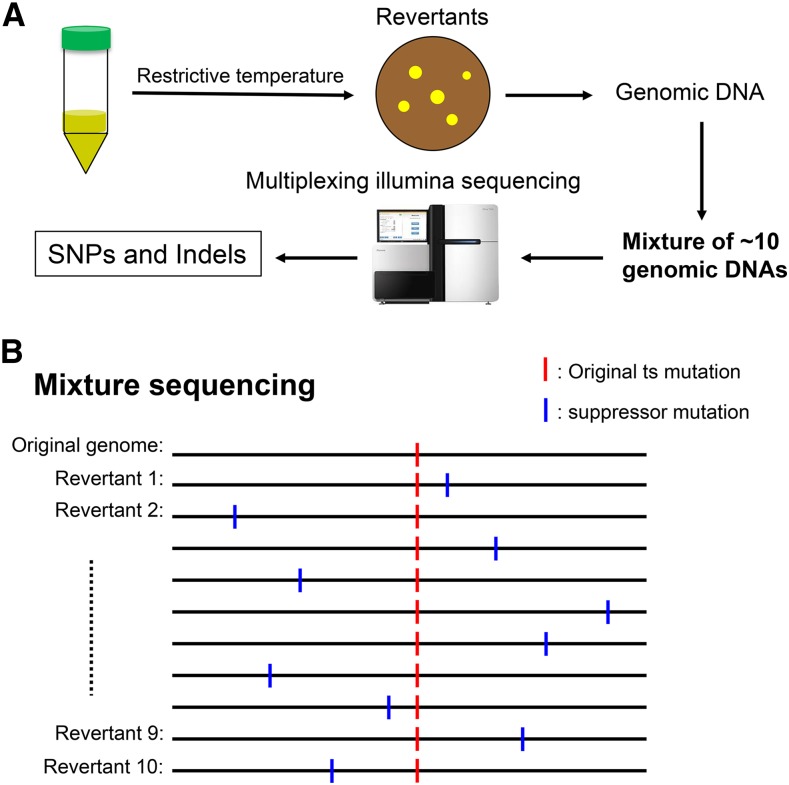
Spontaneous suppressor screen and mixture sequencing strategy. (A) Outline of the spontaneous suppressor screen and mixture next-generation sequencing approach that was developed to identify suppressors compensating for the functions of essential genes. The next-generation sequencing of DNA mixtures method described here is suitable for organisms with small genomes. It enables sequencing of 100 revertant genomes in one lane of Hisequation 2000 paired-end sequencing with only 10 genomic DNA mixtures handled. (B) Schematic explanation of the principle to identify responsible suppressors: 10 revertants of same ts mutant were mixed in equal amounts before library construction; therefore, the allele frequency of the original ts mutation is 100%, while suppressor allele frequencies are ∼10%.

To identify suppressors of ts mutants comprehensively, easily, and cost-effectively, we developed a spontaneous suppressor screening technique, followed by next-generation sequencing of suppressor genomic DNA mixtures (Figure 1A). The technique involved the following steps:

1. Background homogenization. The ts mutants we selected were originally generated by N-methyl-N′-nitrosoguanidine (NTG) mutagenesis. Accordingly, they also have other mutations in addition to the mutation responsible for the ts phenotypes. To exclude possible effects of these silent mutations on suppressor identification, we reintegrated all responsible ts mutations into the *972h^−^* wild-type strain at their endogenous loci under their native promoters using site-directed mutagenesis.
2. Revertant isolation. Newly constructed ts mutants with wild-type background were cultured in YPD liquid medium at 26° (permissive temperature) overnight. Then, cells with spontaneous mutations that occurred in some of them during replication were grown on YPD plates at a concentration of 1 × 10^6^–1 × 10^7^ cells per plate. For each ts mutant, several restrictive temperatures were tested. Then, we selected the temperature with a revertant frequency between 1 × 10^−6^ and 1 × 10^−8^ for screening. This is because we found that, if the revertant frequency is <1 × 10^−8^, suppressors tend to be intragenic (such as when we performed suppressor screen for *cut1-L739S*, a Cut1 separase ts mutant, at restrictive temperature 36°, revertant frequency was ∼1 × 10^−8^, all suppressors are intragenic and surrounding the original L739S mutation. When we performed suppressor screen at restrictive temperature 33°, revertant frequency was ∼1 × 10^−6^, we got multiple instructive extragenic suppressors. Xu *et al.*, personal communication). If the revertant frequency is >1 × 10^−6^, we suppose that suppression might be weak and the genetic interactions might be far; therefore, it might be difficult to understand the mechanism behind the genetic interactions. After incubation at the selected restrictive temperature for 4 d, colonies (revertants containing the original ts mutation and suppressor mutations) were selected for sequencing library construction.
3. Mixture sequencing. Since all revertants of the same ts mutant contain the original ts mutation, and differ by only one suppressor mutation, we mixed genomic DNA of 10 revertants of the same ts mutant in equal proportions and used this genomic DNA mixture to construct a library for sequencing. Then, each DNA mixture was barcoded differently so as to distinguish it, and pooled together for one lane of Hisequation 2000 paired-end sequencing.
4. Candidate suppressor identification. Sequencing reads were mapped to the *S. pombe* reference genome using Novoalign (http://www.novocraft.com), and mutant alleles were identified with SNVer (Wei *et al.* 2011). Except for silent mutations existing in the original *972h^−^* wild-type strain (common mutations), ideally ≤10 suppressor mutations, in addition to the original ts mutation should have been identified in each mixture. The allele frequency of the original ts mutation (and silent mutations existing in the original wild-type strain) was 100%. Since the DNA mixture used for sequencing was a pool of genomic DNA from 10 revertants, the frequency of each suppressor allele was ∼10% (Figure 1B). Candidate suppressors were selected based on the criteria above.
5. Suppressor filtration. Genes with ≥2 independent mutations, or more than two independent mutations in genes involved in same complex, were manually selected as probable suppressors for further analysis.

### Suppressor screen summary

By using the suppressor screen method described above, we identified suppressors for three *S. pombe* ts mutants (*cdc48-G338D*, *eso1-G799D*, and *htb1-G52D*) defective in chromosome segregation at the restrictive temperature (Table 1, Table 2, and Supplemental Material, Table S1 in File S1). Restrictive temperatures 37, 37, and 36° were selected for *cdc48-G338D*, *eso1-G799D*, and *htb1-G52D* respectively for suppressor screen, revertant frequencies at these conditions were all ∼1 × 10^−6^. In total, we sequenced 122 revertants (30 from *cdc48-G338D*, 40 from *eso1-G799D*, and 52 from *htb1-G52D*) with an average coverage for each genome ∼30-fold/bp, and obtained 49 distinct suppressors (19 for *cdc48-G338D*, 14 for *eso1-G799D*, and 16 for *htb1-G52D*).

**Table 2:** Summary of the conditions used for screens and the results obtained

Gene	ts Mutant	Mutation Site	Temperature Used (°)	Cells Plated	Revertants Obtained	Revertants Sequenced	Distinct Suppressors Obtained
cdc48	*cdc48-353*	G338D	37	3.8 × 10^8^	358	30	19
eso1	*eso1-H17*	G799D	37	6 × 10^8^	756	40	14
htb1	*htb1-72*	G52D	36	4.5 × 10^7^	56	52	16

### Cdc48 suppressors

Cdc48/VCP/p97 is an AAA ATPase involved in a variety of cellular processes, including cell cycle progression (Moir *et al.* 1982), proteasome-mediated degradation (Hershko and Ciechanover 1998), and chromosome segregation (Yuasa *et al.* 2004; Ikai and Yanagida 2006). The *cdc48-G338D* ts mutant was isolated through screening for mutants that were suppressed by elevated protease Cut1/separase gene dosage (Yuasa *et al.* 2004).

**Figure 2:**
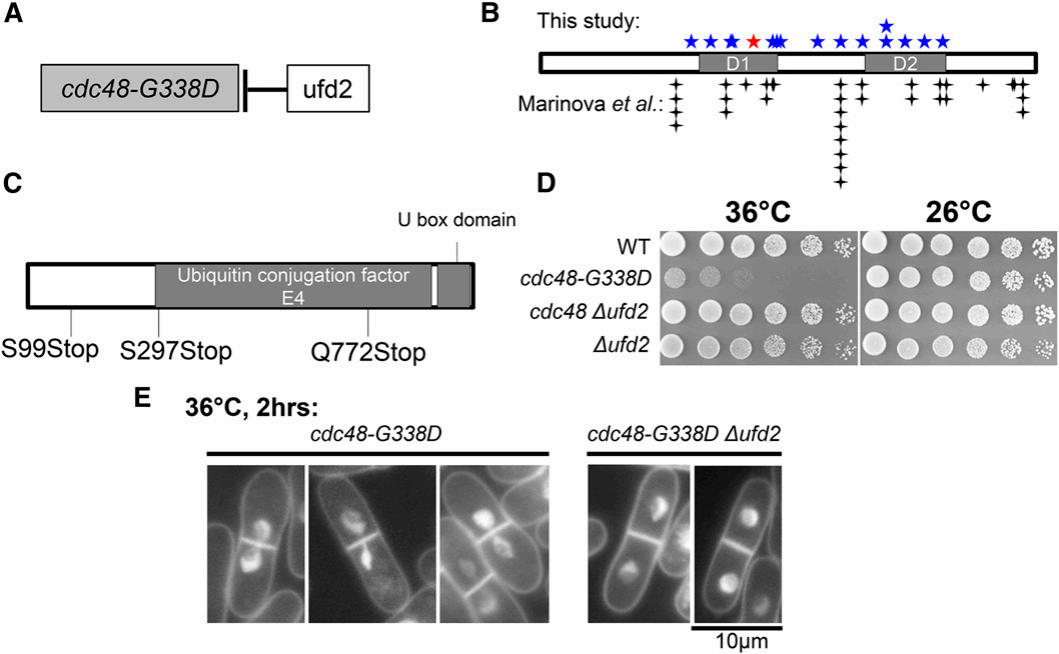
Loss of an E4 multi-ubiquitination enzyme, Ufd2, suppresses *cdc48*. (A) Extragenic suppressors of *cdc48-G338D* were located in *ufd2*. Ufd2 is an E4 multi-ubiquitination enzyme associated with Cdc48. (B) Distribution of *cdc48-G338D* intragenic suppressors identified in this study and comparison with those identified by Marinova *et al.* (2015); the majority of them are located in its ATPase domains (D1 and D2). (C) Domains in Ufd2 and distribution of the three nonsense mutations in Ufd2, all of which may cause loss of the C-terminal E4 activity of Ufd2. (D) Spot test results using wild-type, the single ts mutant *cdc48-G338D*, the double mutant *cdc48-G338D Δufd2*, and the single mutant *Δufd2*. *Δufd2* completely suppressed the ts phenotype of *cdc48-G338D* at 36°, which confirmed the suppressor screening results. (E) The aberrant mitotic phenotype of *cdc48-G338D* was suppressed by *Δufd2* too. Mutant cells were cultured at 36° for 2 hr, stained with DAPI (4′,6-diamidino-2-phenylindole, a fluorescent probe for DNA), and were observed under a fluorescence microscope.

Mutations in *cdc48* itself (intragenic suppressors) and mutations in *ufd2* were isolated as *cdc48-G338D* suppressors in this study (Figure 2, A and B and Table S2 in File S1). The distribution pattern of intragenic *cdc48-G338D* suppressors isolated in the current study is similar to those isolated by Marinova *et al.* (2015). The majority are located in its ATPase domains (D1 and D2, Figure 2B). The original mutation G338D is located in the D1 domain (Figure 2B); therefore, intragenic suppressors in the Cdc48 D1 domain might rescue *cdc48-G338D* through protein conformational changes. Ufd2 is an E4 ubiquitin chain-elongation enzyme associated with Cdc48 to ligate additional ubiquitin moieties to preformed ubiquitin conjugates, so as to promote degradation (Koegl *et al.* 1999). Ufd2 binds to the second AAA domain (D2) of Cdc48, thereby enabling formation of a Cdc48 complex that contains Ufd2 (Richly *et al.* 2005; Rumpf and Jentsch 2006). The association of Ufd2 with Cdc48 might be disrupted in the intragenic suppressors in the Cdc48 D2 domain. All *ufd2* mutations identified in this study are nonsense mutations or insertions that cause the loss of its E4 ubiquitin chain-elongating activity (Figure 2C). *cdc48-G338D Δufd2* confirmed the suppression of *cdc48-G338D* by suppressor mutations in *ufd2* (Figure 2D). *cdc48-G338D* exhibited chromosome segregation defects highly similar to that observed in separase/Cut1 ts mutants at 36° (Yuasa *et al.* 2004), *Δufd2* rescued the aberrant mitotic phenotype of *cdc48-G338D* (Figure 2E).

### Eso1 suppressors

**Figure 3:**
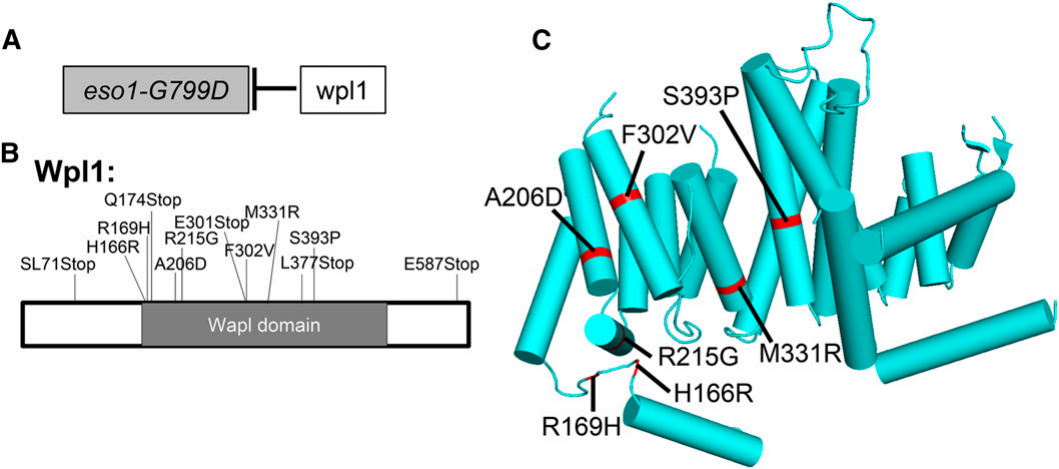
Suppression of a sister chromatid cohesion-defective mutant, *eso1-G799D*, by mutations in cohesin releaser, Wpl1. (A) Application of the mixture sequencing strategy to *eso1-G799D* identified extragenic suppressors in *wpl1*. (B) Distribution of *eso1-G799D* suppressor mutations in Wpl1. Mutations are enriched in the Wapl domain of Wpl1. (C) Localization of Wpl1 missense mutations (which are conserved) in the structure of Wpl1 human homolog Wapl (Ouyang *et al.* 2013).

Eso1 acetylates the Psm3/SMC3 subunit of cohesin at the two conserved lysines, K105 and K106 (corresponding to K112 and K113 in budding yeast), located in the cohesin head domain (Rolef Ben-Shahar *et al.* 2008; Unal *et al.* 2008; Rowland *et al.* 2009; Feytout *et al.* 2011). This acetylation is essential for sister chromatid segregation (Skibbens *et al.* 1999). We isolated spontaneous suppressors of *eso1-G799D* originally isolated by Tanaka *et al.* (2000). *eso1-G799D* suppressors were found only in *wpl1* (Figure 3A and Table S3 in File S1). It is well-known that *Δeso1* is inviable, but the double mutant *Δeso1 Δwpl1* is viable (Feytout *et al.* 2011). Hence, our results are consistent with previous findings. As Wpl1 is thought to be the cohesin releaser (Gandhi *et al.* 2006; Kueng *et al.* 2006), sister chromatid cohesion is more stable in the double mutant. Suppressors identified in Wpl1 were mainly mapped to its Wapl domain (Figure 3B), indicating that rescue of *eso1-G799D* by *wpl1* mutations is caused by the loss of the Wapl domain function of Wpl1. Localization of Wpl1 missense mutations in the structure of the Wpl1 human homolog, Wapl (Ouyang *et al.* 2013), is shown in Figure 3C.

### H2B suppressors

**Figure 4:**
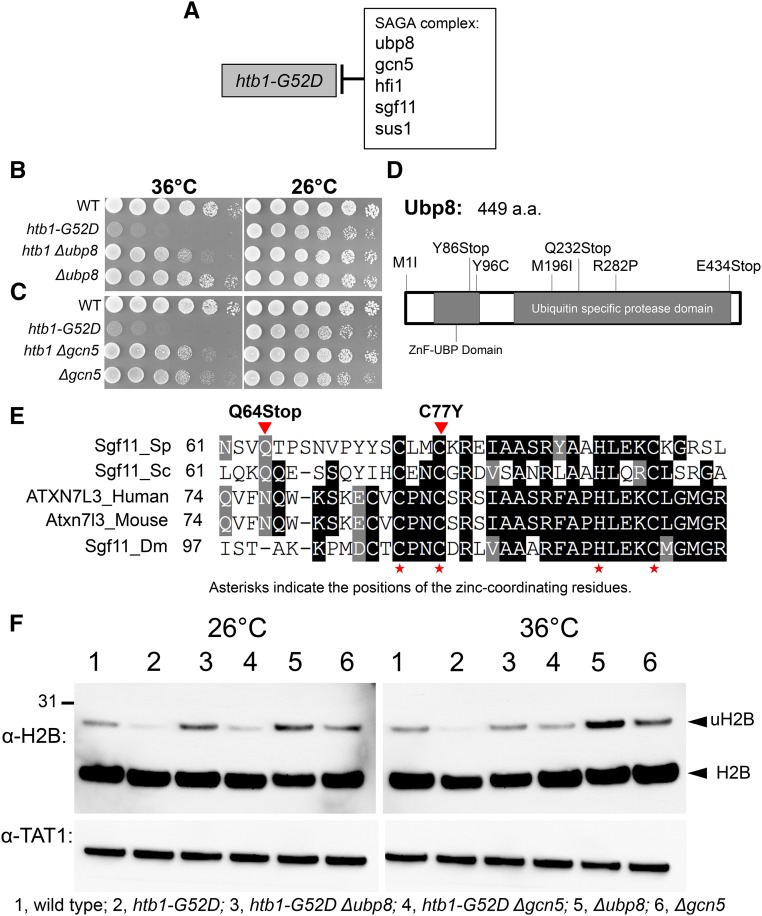
Deubiquitination of H2B by the SAGA complex destabilizes H2B. (A) Extragenic suppressors of *htb1-G52D*, identified by suppressor screening followed by mixture sequencing. All suppressors were mapped to SAGA complex genes. (B) and (C) *htb1-G52D* was crossed with *Δubp8* and *Δgcn5* to produce double mutants. Spot test results indicated that *htb1-G52D* was rescued by *Δubp8* and *Δgcn5*. (D) Almost all *htb1-G52D* single-amino acid-substitution suppressors in Ubp8 were located in the two conserved domains: the ZnF-UBP Domain (ubiquitin-binding domain) and the ubiquitin-specific protease domain, both of which are required for the deubiquitination activity of the SAGA complex. (E) Alignment of Sgf11 among various species and localization of the two *htb1-G52D* suppressors in Sgf11. Sgf11 binds the H2A/H2B heterodimer via its zinc-finger domain (ZnF domain, the positions of the zinc-coordinating residues are indicated by asterisks). The two Sgf11 point mutations (Q64Stop and C77Y) are mapped to its ZnF domain; therefore, they may decrease H2B deubiquitination by the SAGA complex. (F) Immunoblot patterns using a polyclonal antibody against histone H2B (anti-H2B). Wild type, *htb1-G52D*, *htb1-G52D Δubp8*, *htb1-G52D Δgcn5*, *Δubp8*, and *Δgcn5* were cultured first at 26°, and then shifted to 36° for 5 hr. The upper band is mono-ubiquitinated H2B (uH2B) and the lower band is H2B.

*htb1-G52D* is a histone H2B ts mutant. It exhibits a gene-silencing defect in heterochromatic regions and lagging chromosomes in anaphase (Maruyama *et al.* 2006). *htb1-G52D* suppressors were located in Spt-Ada-Gcn5-Acetyl transferase (SAGA) complex genes (Figure 4A and Table S4 in File S1). The SAGA complex is a multiprotein complex that can be divided into five distinct functional modules: a recruitment module (Tra1), an acetylation module (Gcn5, Ada2, and Ngg1/Ada3), a TBP interaction unit (Spt3 and Spt8), a Dub module (Ubp8, Sus1, Sgf11, and Sgf73), and an architecture unit (Spt7, Spt20, Hfi1/Ada1, Taf5, Taf6, Taf9, and Taf12) (Koutelou *et al.* 2010). Among the protein products of the five genes identified, Ubp8, Sgf11, and Sus1 are in the deubiquitination (DUB) module. Gcn5 is in the histone acetylation (HAT) module, and Hfi1/Ada1 is in the architecture module (Figure 4A). The double mutants, *htb1-G52D Δubp8* and *htb1-G52D Δgcn5*, confirmed the genetic suppression events (Figure 4, B and C). Histone H2B is mono-ubiquitinated at K119 in fission yeast (corresponding to K123 in budding yeast and K120 in humans) and Ubp8 in the deubiquitinating (DUB) module of the SAGA complex is the deubiquitinase responsible for catalyzing histone H2B deubiquitination (Henry *et al.* 2003; Daniel *et al.* 2004). Importantly, Ubp8 alone does not possess H2B deubiquitinating activity, unless it is integrated into the SAGA complex (Köhler *et al.* 2010). Sgf11, Sus1, and Sgf73 are required for tethering Ubp8 to the SAGA complex and serve, in part, as scaffolds that help to maintain the Ubp8 catalytic domain in an active conformation (Lee *et al.* 2005; Köhler *et al.* 2006, 2008; Samara *et al.* 2012; Yan and Wolberger 2015). Sgf11 activates Ubp8, and its ZnF domain plays a key role in this activation (Han *et al.* 2014).

Ubp8 possesses a ubiquitin-binding domain (ZnF-UBP Domain) at its N-terminus and a “Ubiquitin-specific protease domain” at its C-terminus. Almost all *htb1-G52D* single-amino acid-substitution suppressors in Ubp8 were located in these two conserved domains (Figure 4D). The Ubp8 ZnF-UBP domain binds Sgf11 N-terminal helix by Sus1 (Köhler *et al.* 2010). Mutations in this domain (Y86Stop and Y96C) may cause a weakened association of Ubp8 with the SAGA complex. While the “Ubiquitin-specific protease domain” is another domain of Ubp8, it is the catalytic domain performing the deubiquitination, therefore mutations in this domain may cause Ubp8 to lose its deubiquitination activity.

Recent structural information indicates that the DUB module binds to the acidic patch of the H2A/H2B heterodimer via the Sgf11 ZnF domain (Zinc finger domain) (Morgan *et al.* 2016). The ZnF domain of Sgf11 is a small CCHC-type zinc-finger domain at the very C-terminal end. Two Sgf11 point mutations (Q64Stop and C77Y) are mapped to its ZnF domain (Figure 4E), which may decrease the deubiquitination activity of the DUB module by decreasing its association with the H2A/H2B heterodimer.

To support that SAGA complex deubiquitinates H2B, we examined the H2B mono-ubiquitination level in SAGA-complex mutants, using a polyclonal antibody against histone H2B (anti-H2B) (Figure 4F). The H2B mono-ubiquitination levels (upper band) decreased in the original ts mutant *htb1-G52D*, but were recovered in *htb1-G52D Δubp8*, and *htb1-G52D Δgcn5* double mutants. The H2B mono-ubiquitination levels were further enhanced in *Δubp8* and *Δgcn5* single mutants.

Taken together, suppression of *htb1-G52D* by SAGA-complex mutations is caused by loss of the deubiquitination activity of the complex. In addition, since many mutations in other modules (Gcn5 in the histone acetylation module and Hfi1/Ada1 in the architecture module) were identified, these modules may also be required for the deubiquitination activity of the SAGA complex, mediated by its DUB module.

## Discussion

Genetic interactions have been mapped extensively, not only in budding yeast (Costanzo *et al.* 2010, 2016; van Leeuwen *et al.* 2016), but also in fission yeast (Roguev *et al.* 2007, 2008). In this study, we started with established ts mutants and intended to systematically identify suppressors for the targeted ts mutants. Therefore, we established a method employing genomic DNA mixtures followed by next-generation sequencing to efficiently identify suppressors of ts mutants at low cost, regardless of whether the revertants are cold sensitive. This method is applicable to budding yeast too, since a ts collection of 787 ts strains is available, with all alleles integrated into their native genomic loci in the S288C common reference strain (Li *et al.* 2011).

Genetic suppression provides a way to identify genes that when mutated can compensate for defects of an original mutation. Extragenic suppressor genes are functionally related to the original gene. They encode either members of the same pathway/complex or proteins regulating the activity, stability, or degradation of the original protein (van Leeuwen *et al.* 2016). A variety of suppressors in the same gene enable us to determine which function of the encoded protein is defective (based on the function of the domain in which suppressors are enriched). This is very helpful in predicting mechanisms underlying the genetic suppression.

**Figure 5:**
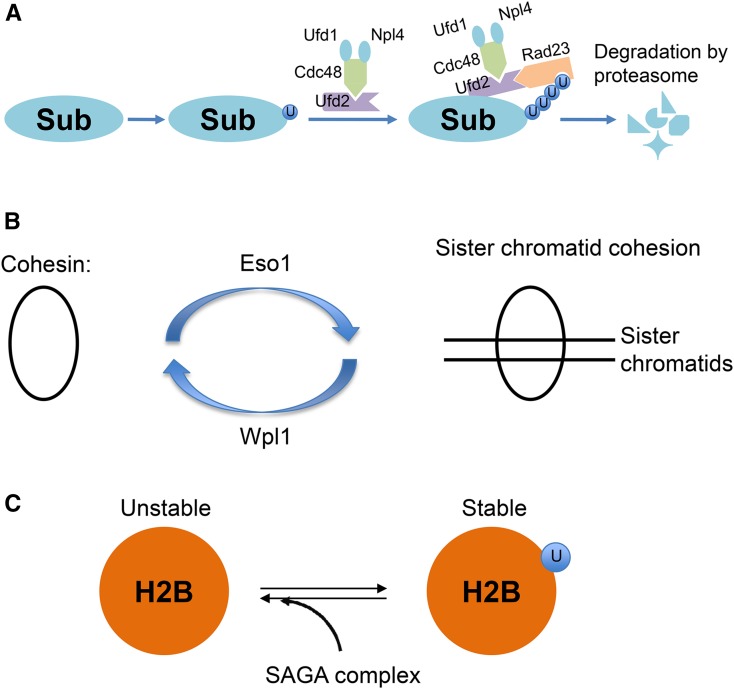
Mechanism of genetic suppression. (A) Ufd2 attaches additional ubiquitin molecules to Cdc48-bound mono- (oligo-) ubiquitinated substrates to promote protein degradation. Protein degradation might be facilitated in the *cdc48-G338D* mutant, but compromised by *ufd2* mutations. The Cdc48-Ufd2 part is adapted from Baek *et al.* (2011). (B) Eso1 and Wpl1 act on cohesin. Eso1 is required for establishment of sister chromatid cohesion and Wpl1 enhances cohesin release from chromatin. (C) H2B is stabilized by mono-ubiquitination and ubiquitination is removed from H2B by the deubiquitination activity of the SAGA complex.

Suppression mechanisms illustrated here for the three ts mutants are distinct. Ufd2 attaches additional ubiquitin molecules to Cdc48-bound, mono- (or oligo-) ubiquitinated substrates, targeting them for degradation (Koegl *et al.* 1999) (Figure 5A). Protein degradation might be facilitated in *cdc48-G338D* mutant, as levels of Cut1 and Cut2 (two potential Cdc48 substrates) are diminished in the *cdc48-G338D* mutant (Ikai and Yanagida 2006). Loss of Ufd2, which may slow degradation of Cdc48 substrates, such as Cut1 and Cut2, therefore compromises the growth defects of *cdc48-G338D* at the restrictive temperature. Eso1 is required for establishment of sister chromatid cohesion, while Wpl1 is a cohesin-releasing factor. The sister chromatid cohesion defect in *eso1-G799D* may be overcome by loss of a cohesin-releasing factor, Wpl1. Sister chromatid cohesion is regulated by the opposing effects of Eso1 and Wpl1, and sister chromatid cohesion is balanced when Eso1 and Wpl1 are both defective (Figure 5B). H2B is a substrate of the SAGA complex deubiquitination module and H2B is stabilized by mono-ubiquitination (Maruyama *et al.* 2006); therefore, suppression of *htb1-G52D* by SAGA complex mutations was due to stabilization of H2B (Figure 5C).

The cost-effective suppressor screen method described here has enabled us to sequence >3400 spontaneous revertants of ∼50 ts mutants isolated in this laboratory. The resulting ∼900 independent suppressor mutations, which met the standards described above, have been identified and will be described elsewhere.

## Supplementary Material

Supplemental material is available online at www.g3journal.org/lookup/suppl/doi:10.1534/g3.118.200048/-/DC1.
