# Supplementary material for: Whole-Genome Sequencing of Suppressor DNA Mixtures Identifies Pathways That Compensate for Chromosome Segregation Defects in Schizosaccharomyces pombe
Source: G3 (Bethesda). 2018 Jan 19;8(3):1031–8. doi: 10.1534/g3.118.200048 (PMC5844291; doi:10.1534/g3.118.200048)
Supplement: Supplementary file 1 [file 1031FileS1.pptx]

## Slide 1
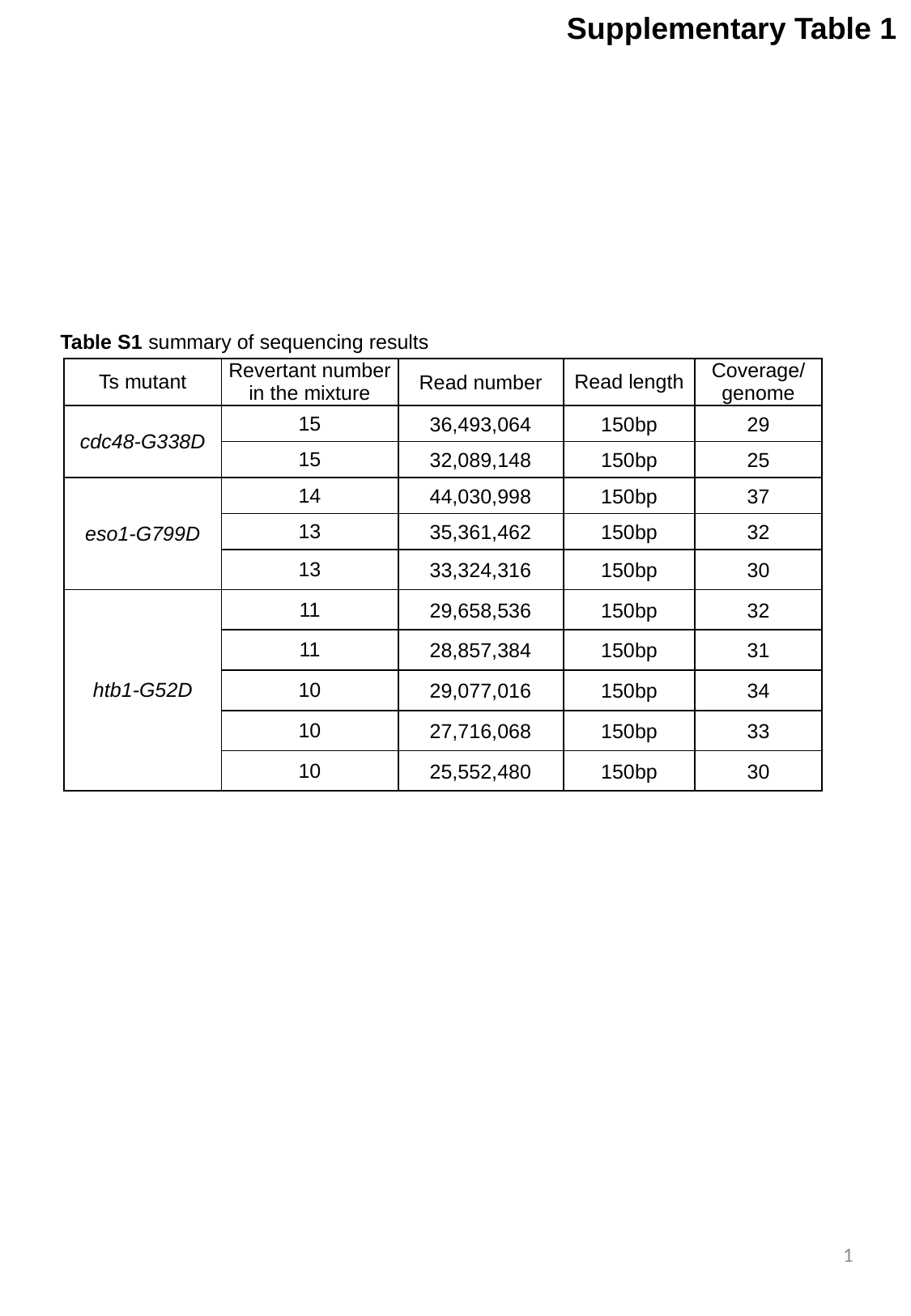

Supplementary Table 1
Table S1 summary of sequencing results
| Ts mutant | Revertant number in the mixture | Read number | Read length | Coverage/genome |
| --- | --- | --- | --- | --- |
| cdc48-G338D | 15 | 36,493,064 | 150bp | 29 |
| | 15 | 32,089,148 | 150bp | 25 |
| eso1-G799D | 14 | 44,030,998 | 150bp | 37 |
| | 13 | 35,361,462 | 150bp | 32 |
| | 13 | 33,324,316 | 150bp | 30 |
| htb1-G52D | 11 | 29,658,536 | 150bp | 32 |
| | 11 | 28,857,384 | 150bp | 31 |
| | 10 | 29,077,016 | 150bp | 34 |
| | 10 | 27,716,068 | 150bp | 33 |
| | 10 | 25,552,480 | 150bp | 30 |
1

## Slide 2
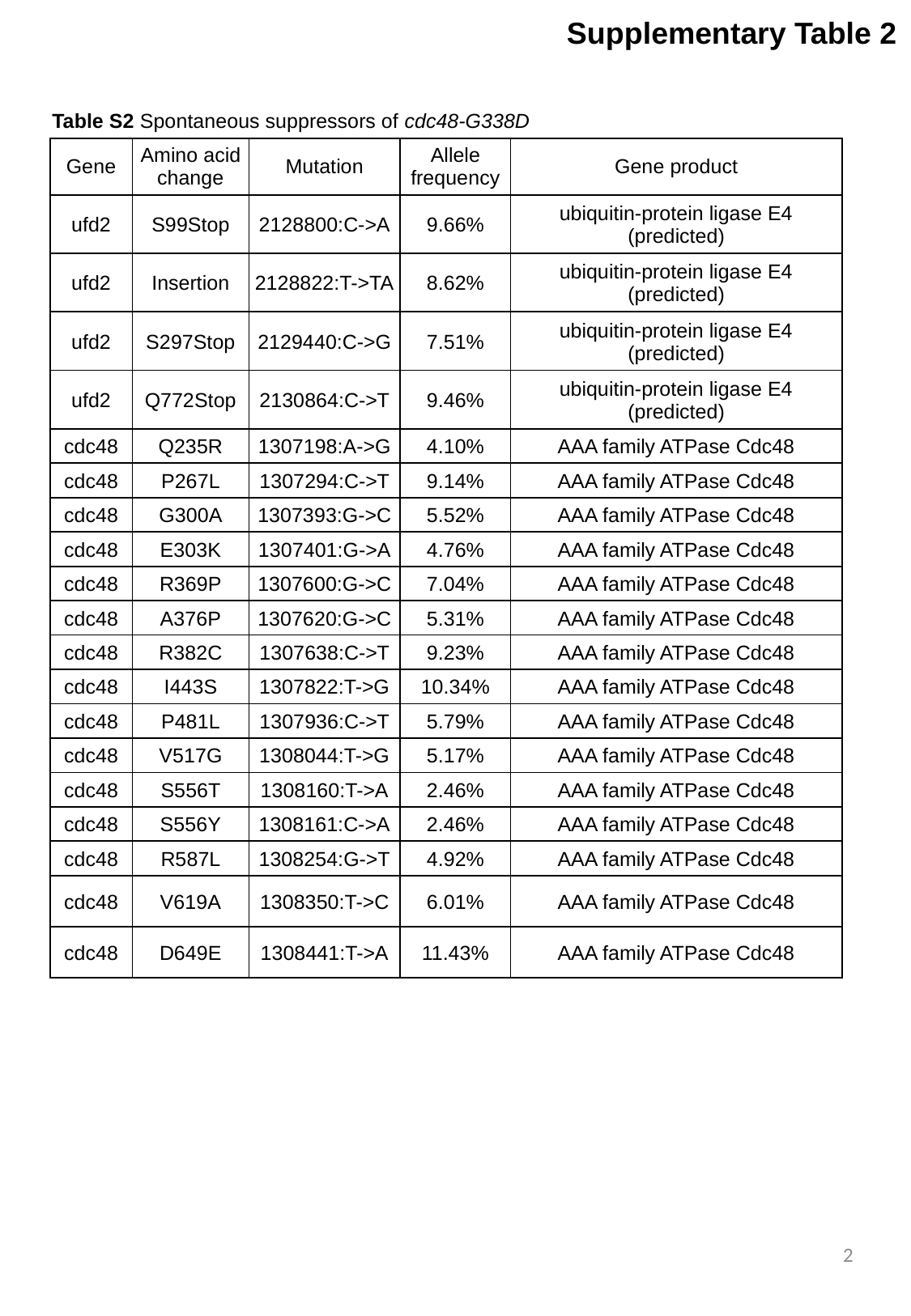

Supplementary Table 2
Table S2 Spontaneous suppressors of cdc48-G338D
| Gene | Amino acid change | Mutation | Allele frequency | Gene product |
| --- | --- | --- | --- | --- |
| ufd2 | S99Stop | 2128800:C->A | 9.66% | ubiquitin-protein ligase E4 (predicted) |
| ufd2 | Insertion | 2128822:T->TA | 8.62% | ubiquitin-protein ligase E4 (predicted) |
| ufd2 | S297Stop | 2129440:C->G | 7.51% | ubiquitin-protein ligase E4 (predicted) |
| ufd2 | Q772Stop | 2130864:C->T | 9.46% | ubiquitin-protein ligase E4 (predicted) |
| cdc48 | Q235R | 1307198:A->G | 4.10% | AAA family ATPase Cdc48 |
| cdc48 | P267L | 1307294:C->T | 9.14% | AAA family ATPase Cdc48 |
| cdc48 | G300A | 1307393:G->C | 5.52% | AAA family ATPase Cdc48 |
| cdc48 | E303K | 1307401:G->A | 4.76% | AAA family ATPase Cdc48 |
| cdc48 | R369P | 1307600:G->C | 7.04% | AAA family ATPase Cdc48 |
| cdc48 | A376P | 1307620:G->C | 5.31% | AAA family ATPase Cdc48 |
| cdc48 | R382C | 1307638:C->T | 9.23% | AAA family ATPase Cdc48 |
| cdc48 | I443S | 1307822:T->G | 10.34% | AAA family ATPase Cdc48 |
| cdc48 | P481L | 1307936:C->T | 5.79% | AAA family ATPase Cdc48 |
| cdc48 | V517G | 1308044:T->G | 5.17% | AAA family ATPase Cdc48 |
| cdc48 | S556T | 1308160:T->A | 2.46% | AAA family ATPase Cdc48 |
| cdc48 | S556Y | 1308161:C->A | 2.46% | AAA family ATPase Cdc48 |
| cdc48 | R587L | 1308254:G->T | 4.92% | AAA family ATPase Cdc48 |
| cdc48 | V619A | 1308350:T->C | 6.01% | AAA family ATPase Cdc48 |
| cdc48 | D649E | 1308441:T->A | 11.43% | AAA family ATPase Cdc48 |
2

## Slide 3
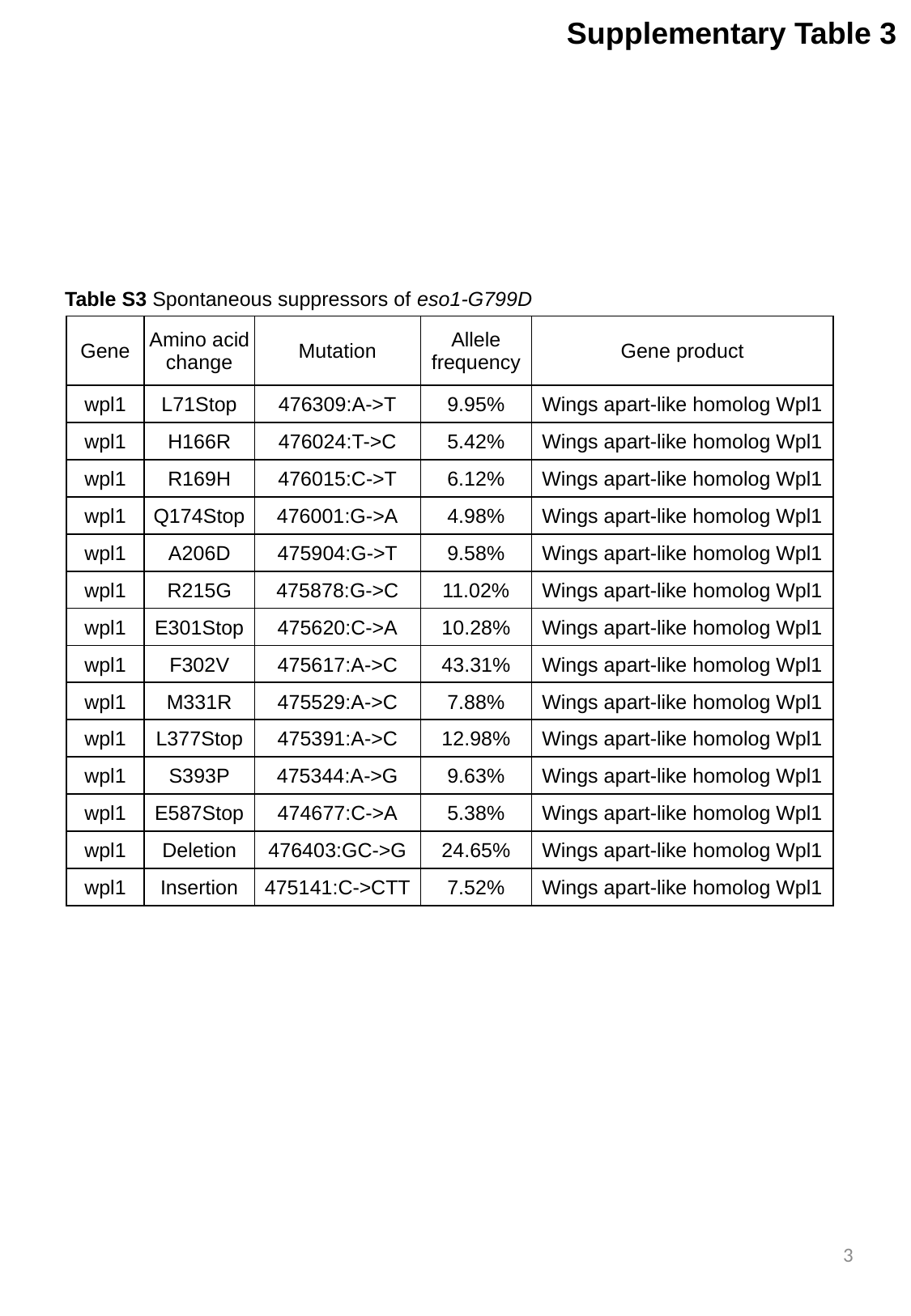

Supplementary Table 3
Table S3 Spontaneous suppressors of eso1-G799D
| Gene | Amino acid change | Mutation | Allele frequency | Gene product |
| --- | --- | --- | --- | --- |
| wpl1 | L71Stop | 476309:A->T | 9.95% | Wings apart-like homolog Wpl1 |
| wpl1 | H166R | 476024:T->C | 5.42% | Wings apart-like homolog Wpl1 |
| wpl1 | R169H | 476015:C->T | 6.12% | Wings apart-like homolog Wpl1 |
| wpl1 | Q174Stop | 476001:G->A | 4.98% | Wings apart-like homolog Wpl1 |
| wpl1 | A206D | 475904:G->T | 9.58% | Wings apart-like homolog Wpl1 |
| wpl1 | R215G | 475878:G->C | 11.02% | Wings apart-like homolog Wpl1 |
| wpl1 | E301Stop | 475620:C->A | 10.28% | Wings apart-like homolog Wpl1 |
| wpl1 | F302V | 475617:A->C | 43.31% | Wings apart-like homolog Wpl1 |
| wpl1 | M331R | 475529:A->C | 7.88% | Wings apart-like homolog Wpl1 |
| wpl1 | L377Stop | 475391:A->C | 12.98% | Wings apart-like homolog Wpl1 |
| wpl1 | S393P | 475344:A->G | 9.63% | Wings apart-like homolog Wpl1 |
| wpl1 | E587Stop | 474677:C->A | 5.38% | Wings apart-like homolog Wpl1 |
| wpl1 | Deletion | 476403:GC->G | 24.65% | Wings apart-like homolog Wpl1 |
| wpl1 | Insertion | 475141:C->CTT | 7.52% | Wings apart-like homolog Wpl1 |
3

## Slide 4
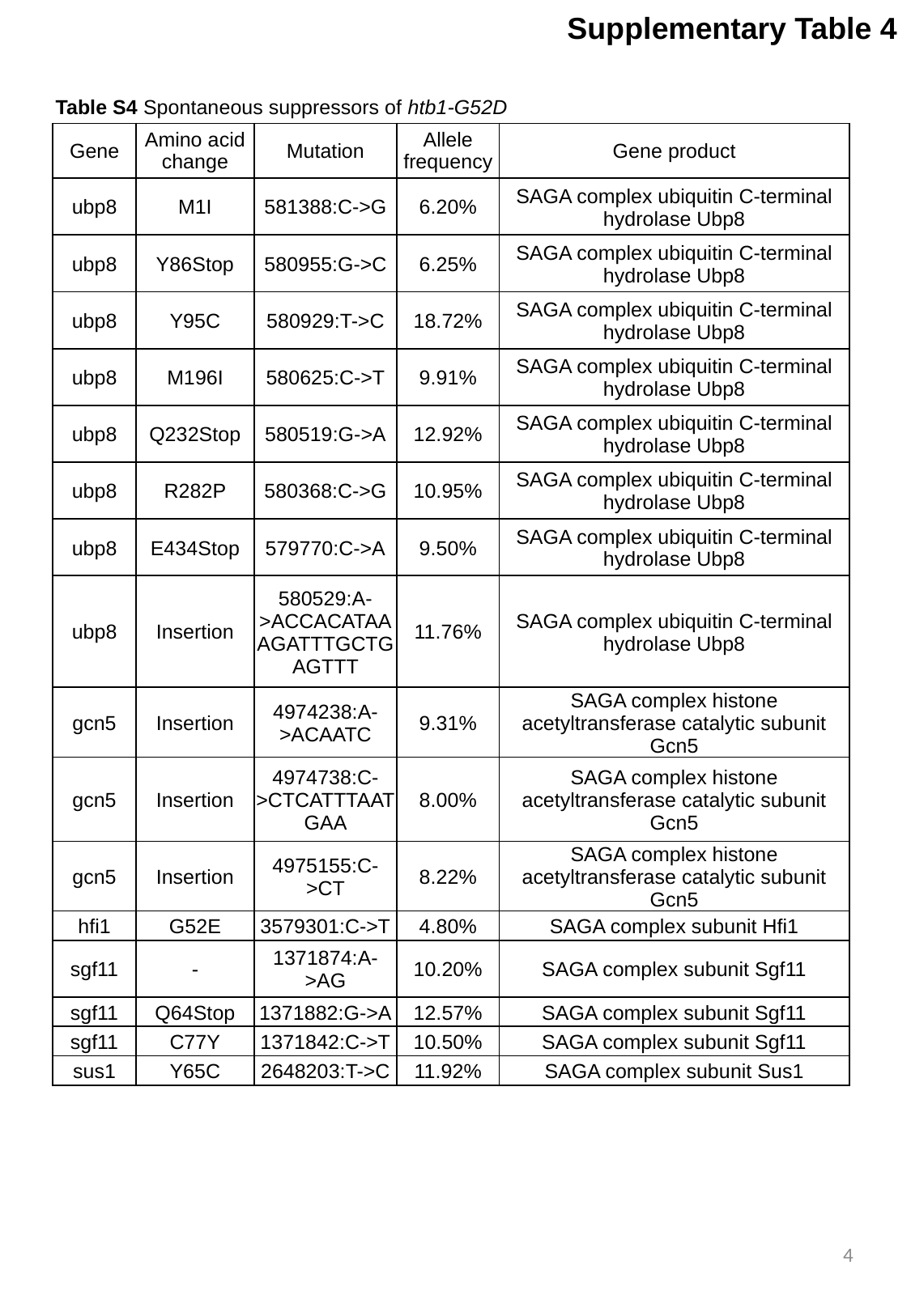

Supplementary Table 4
Table S4 Spontaneous suppressors of htb1-G52D
| Gene | Amino acid change | Mutation | Allele frequency | Gene product |
| --- | --- | --- | --- | --- |
| ubp8 | M1I | 581388:C->G | 6.20% | SAGA complex ubiquitin C-terminal hydrolase Ubp8 |
| ubp8 | Y86Stop | 580955:G->C | 6.25% | SAGA complex ubiquitin C-terminal hydrolase Ubp8 |
| ubp8 | Y95C | 580929:T->C | 18.72% | SAGA complex ubiquitin C-terminal hydrolase Ubp8 |
| ubp8 | M196I | 580625:C->T | 9.91% | SAGA complex ubiquitin C-terminal hydrolase Ubp8 |
| ubp8 | Q232Stop | 580519:G->A | 12.92% | SAGA complex ubiquitin C-terminal hydrolase Ubp8 |
| ubp8 | R282P | 580368:C->G | 10.95% | SAGA complex ubiquitin C-terminal hydrolase Ubp8 |
| ubp8 | E434Stop | 579770:C->A | 9.50% | SAGA complex ubiquitin C-terminal hydrolase Ubp8 |
| ubp8 | Insertion | 580529:A->ACCACATAAAGATTTGCTGAGTTT | 11.76% | SAGA complex ubiquitin C-terminal hydrolase Ubp8 |
| gcn5 | Insertion | 4974238:A->ACAATC | 9.31% | SAGA complex histone acetyltransferase catalytic subunit Gcn5 |
| gcn5 | Insertion | 4974738:C->CTCATTTAATGAA | 8.00% | SAGA complex histone acetyltransferase catalytic subunit Gcn5 |
| gcn5 | Insertion | 4975155:C->CT | 8.22% | SAGA complex histone acetyltransferase catalytic subunit Gcn5 |
| hfi1 | G52E | 3579301:C->T | 4.80% | SAGA complex subunit Hfi1 |
| sgf11 | - | 1371874:A->AG | 10.20% | SAGA complex subunit Sgf11 |
| sgf11 | Q64Stop | 1371882:G->A | 12.57% | SAGA complex subunit Sgf11 |
| sgf11 | C77Y | 1371842:C->T | 10.50% | SAGA complex subunit Sgf11 |
| sus1 | Y65C | 2648203:T->C | 11.92% | SAGA complex subunit Sus1 |
4
